# Differences in the pathogenicity and molecular characteristics of fowl adenovirus serotype 4 epidemic strains in Guangxi Province, southern China

**DOI:** 10.3389/fmicb.2024.1428958

**Published:** 2024-06-27

**Authors:** You Wei, Zhiqin Xie, Zhixun Xie, Xianwen Deng, Xiaofeng Li, Liji Xie, Qing Fan, Yanfang Zhang, Sheng Wang, Hongyu Ren, Lijun Wan, Sisi Luo, Meng Li

**Affiliations:** ^1^Guangxi Key Laboratory of Veterinary Biotechnology, Guangxi Veterinary Research Institute, Nanning, Guangxi, China; ^2^Key Laboratory of China (Guangxi)-ASEAN Cross-Border Animal Disease Prevention and Control, Ministry of Agriculture and Rural A-airs of China, Nanning, Guangxi, China

**Keywords:** fowl adenovirus serotype 4, wild-type low-virulence strain, pathogenicity difference, molecular characteristics, Guangxi Province, GX2019-014

## Abstract

Starting in 2015, the widespread prevalence of hydropericardium-hepatitis syndrome (HHS) has led to considerable financial losses within China’s poultry farming industry. In this study, pathogenicity assessments, whole-genome sequencing, and analyses were conducted on 10 new isolates of the novel genotype FAdV-4 during a HHS outbreak in Guangxi Province, China, from 2019 to 2020. The results indicated that strains GX2019-010 to GX2019-013 and GX2019-015 to GX2019-018 were highly virulent, while strain GX2020-019 exhibited moderate virulence. Strain GX2019-014 was characterized as a wild-type strain with low virulence, displaying no pathogenic effects when 0.5 mL containing 10^6^ TCID_50_ virus was inoculated into the muscle of specific pathogen-free (SPF) chickens at 4 weeks of age, while 10^7^ TCID_50_ and 10^8^ TCID_50_ resulted in mortality rates of 80 and 100%, respectively. The whole genomes of strains GX2019-010 to GX2019-013, GX2019-015 to GX2019-018, and GX2020-019 showed high homology with other Chinese newly emerging highly pathogenic FAdV-4 strains, whereas GX2019-014 was closer to nonmutant strains and shared the same residues with known nonpathogenic strains (B1-7, KR5, and ON1) at positions 219AA and 380AA of the Fiber-2 protein. Our work enriches the research on prevalent strains of FAdV-4 in China, expands the knowledge on the virulence diversity of the novel genotype FAdV-4, and provides valuable reference material for further investigations into the key virulence-associated genetic loci of FAdV-4.

## 1 Introduction

According to the “ICTV Virus Taxonomy Profile: Adenoviridae 2022” released by the International Committee on Taxonomy of Viruses (ICTV), the Adenoviridae family is presently organized into six acknowledged genera (*Aviadenovirus*, *Siadenovirus*, *Atadenovirus*, *Mastadenovirus*, *Ichtadenovirus*, and *Testadenovirus*) based on specific genomic characteristics and molecular phylogenetic analysis. Within the *Aviadenovirus* genus, there are fifteen species (*Fowl Aviadenovirus* A-E, *Turkey Aviadenovirus* B-D, *Falcon Aviadenovirus* A, *Duck Aviadenovirus* B, *Goose Aviadenovirus* A, *Pigeon Aviadenovirus* A-B and *Psittacine Aviadenovirus* B-C) delineated through phylogenetic analysis, genome structure and lack of notable cross-neutralization (Benkő et al., 2022; Chen et al., 2022). The five species (A-E) of *Fowl Aviadenoviruses* included 12 serotypes (Serotype 1 to 8a and 8b to 11) (Mcferran and Smyth, 2000; Marek et al., 2016).

Fowl adenovirus serotype 4 (FAdV-4) belongs to the *Aviadenovirus* C species. It is an unenveloped, linear double-stranded DNA virus with a genome spanning 43 to 46 kb in length. The principal structural proteins found on the capsid include Hexon, the Penton base, Fiber-1, and Fiber-2. The major core shell proteins include pV, pVII, pVIII, and pX. The known nonstructural proteins include 52/55 k, E1A, E1B, E3, E4, and 100 k (Rux et al., 2003). Recent studies have indicated that Hexon creates neutralizing antigenic sites housing clusters of serotype-specific antigenic determinants that are commonly used for phylogenetic analysis (Rux et al., 2003). The presence of penton bases influences viral entry into cells (Fender et al., 2005). Fiber-1 mediates infection through the shaft-knob structure and is a target of the host cell receptor CAR (Wang et al., 2020; Lu et al., 2022). Fiber-2 is a virulence determinant cluster and exhibits good antigenicity, inducing the production of neutralizing antibodies and effectively combating FAdV-4 infection (Schachner et al., 2014; Wang et al., 2018; Quan et al., 2021).

FAdV-4 has been confirmed as the pathogen causing hydropericardium hepatitis syndrome (HHS) (Abe et al., 1998). HHS was initially documented in the Angara region of Pakistan in 1987, with subsequent outbreaks observed in various regions, including Australia, Iraq, Kuwait, India, Japan, and Korea (Dahiya et al., 2002; Choi et al., 2012; Miyaoka et al., 2021; Ishag et al., 2022). Since 2015, sporadic outbreaks have emerged in China and rapidly spread on a large scale within major poultry farming provinces, including Henan, Shandong, Yunnan, Jilin, Guangdong, and Guangxi (Niu et al., 2018; Chen et al., 2019; Rashid et al., 2020). HHS has become among the main emerging diseases in chicks in recent years. Chickens typically develop clinical symptoms, such as fluffy feathers, whitish combs, depression, huddling in corners, diarrhea, and sporadic death, 2–4 days after contracting the virus (Zhao et al., 2015). The onset of evident symptoms and peak mortality occur 3–6 days postinfection; in this phase, chickens pass yellow–green feces, refuse to eat or drink, show cyanotic combs, exhibit lethargy, and eventually die (Kai et al., 2019; Wei et al., 2023). Postmortem examination revealed the accumulation of yellowish fluid in the pericardial sac; hepatomegaly with friable texture, yellow discoloration, and obvious hemorrhagic spots on the liver surface; splenomegaly with congestion; renal hemorrhage; and other typical pathological changes (Mo et al., 2019), which became more pronounced as the disease progressed. This disease causes mortality and growth retardation in both broiler and layer chickens, with clinical mortality rates reaching 20 to 80%. In addition, the immune organs of surviving birds are damaged, leading to immunosuppression in poultry flocks and substantial financial setbacks for the poultry sector (Wei et al., 2023). Therefore, disease prevention and control have become key focuses in poultry farming and clinical settings in recent years.

Since 2016, suspected instances of HHS have been reported in cities such as Nanning, Yulin, Baise, and Qinzhou within Guangxi Province, China (Rashid et al., 2020). In this study, 10 strains of FAdV-4 were isolated from commercial poultry farms and individual poultry farms in Guangxi Province, China, and their pathogenicity in specific pathogen-free (SPF) chickens was assessed. Furthermore, the complete genomes of all 10 isolates were sequenced, and nucleotide and amino acid-level comparisons were conducted with both pathogenic and nonpathogenic strains of FAdV-4 available in GenBank to analyze key virulence-related sites. This study aimed to provide data supporting the epidemiological investigation of FAdV-4 in Guangxi Province and to provide relevant information for the study of virulence determinant sites of FAdV-4.

## 2 Materials and methods

### 2.1 Experimental animals and ethical statement

One thousand SPF chickens utilized to assess the pathogenicity of the FAdV-4 isolates were derived from SPF White Leghorn chicken eggs obtained from Beijing Boehringer Ingelheim Vital Biotechnology Co., Ltd. (Beijing, China) and hatched naturally in an incubator and subsequently reared in negative-pressure SPF isolation facilities until they reached 4 weeks of age. The animal experiments were conducted following approval from the Animal Ethics Committee of the Guangxi Veterinary Research Institute and adhered strictly to their regulations (Approval No. 2019c0406).

### 2.2 Sample collection and PCR detection of FAdV-4

All the isolates were obtained from poultry flocks on chicken farms in the cities of Nanning, Baise, and Yulin in Guangxi Province, China, during the HHS outbreak from 2019 to 2020. The relevant information regarding the sources of these isolates is provided in Table 1. The hearts, livers, spleens, kidneys, lungs, bursa of Fabricius, and pancreatic tissues of the suspected chickens were collected, homogenized separately in phosphate-buffered saline (PBS), subjected to three freeze–thaw cycles, and centrifuged at 10,000 × g for 15 min to obtain the supernatant. Viral DNA was extracted from the supernatant following the instructions of the TransGen Biotech EasyPure Genomic DNA/RNA Kit (TransGen, China). Common PCR detection of FAdV-4 was conducted using specific primers targeting the Hexon gene (Zhang et al., 2016): forward, 5’-CGAGGTCTAT ACCAACACGAGCA-3′; reverse, 5’-TACAGCAGGTTAATGA AGTTATC-3′ (Sangon Biotech, China). The amplification program comprised an initial denaturation at 95°C for 5 min, followed by 30 amplification cycles, each consisting of denaturation at 95°C for 30 s, annealing at 55°C for 30 s, and extension at 72°C for 1 min, with a final extension step at 72°C for 10 min. Gel electrophoresis was performed, and samples with strongly positive bands were selected for virus isolation.

**Table 1:** Information regarding the origins of the isolates.

No.	Name of the isolate	Sampling time	Sampling place	Age of the chicken	Sampling tissue
1	GX2019-010	2019.08.07	GuangXi Province, Nanning City	24	Live
2	GX2019-011	2019.08.07	GuangXi Province, Nanning City	46	Live
3	GX2019-012	2019.08.07	GuangXi Province, Nanning City	53	Live
4	GX2019-013	2019.11.07	GuangXi Province, Baise City	60	Live
5	GX2019-014	2019.11.07	GuangXi Province, Baise City	50	Kidney
6	GX2019-015	2019.11.07	GuangXi Province, Baise City	55	Live
7	GX2019-016	2019.11.12	GuangXi Province, Nanning City	32	Live
8	GX2019-017	2019.04.28	GuangXi Province, Yulin City	45	Live
9	GX2019-018	2019.08.06	GuangXi Province, Baise City	56	Live
10	GX2020-019	2020.09.12	GuangXi Province, Yulin City	42	Live

### 2.3 Primary chicken embryonic liver cell preparation and isolation

The chicken embryos were derived from SPF White Leghorn chicken eggs, which were incubated in an incubator until 15 days of age. Liver tissues were dissected from 15-day-old embryos and minced to approximately 2 mm^3^. After digestion of liver tissue in 0.25% trypsin–EDTA solution for 10 min and gentle pipetting, liver cells were suspended in Dulbecco’s modified Eagle’s medium/nutrient mixture F-12 (DMEM/F12) (Gibco, USA) supplemented with 10% fetal bovine serum (Gibco, USA). The suspension was filtered through a sterile sieve with a 40-μm aperture, and the cell count was adjusted to 10^6^ CEL cells/ml. The cells were cultured in T-25 flasks for approximately 72 h until they formed a monolayer (Wei et al., 2023).

The homogenized supernatant from FAdV-4 positive samples was filtered through a sterile syringe filter (PES) with 0.22 μm pores to remove bacteria. The viral suspension was inoculated into CEL at 2% of the volume of the culture medium, cultured for 96–120 h, and subjected to blind passage for two generations for virus isolation, after which the cell supernatant was collected.

### 2.4 Virus purification and identification

The isolated virus was subjected to plaque purification using CEL cells. The supernatant was diluted from 10^−3^ to 10^−8^ and inoculated onto 6-well cell culture plates containing confluent monolayers of CEL cells. After 1 h of infection, the supernatant was aspirated. The cell surface was then coated with DMEM/F12 supplemented with 2% fetal bovine serum and 1% low melting point agarose (Promega, USA). The cells were incubated at 37°C and 5% CO^2^ for 6 days. Isolated, well-defined, medium-sized individual plaques were selected for three rounds of plaque purification.

The purified isolates were named according to the format ‘Abbreviation of Source Location and Year of Isolation-Purification Order’. After purification, the viruses were propagated in CEL cells on a large scale (600 mL/isolate), followed by aliquoting into cryovials at 2 mL/vial, with approximately 300 vials/isolate, to establish a seed bank. Any vial of viral solution from this batch can represent the entire seed bank. The seed bank was stored at −80°C and used for subsequent indirect immunofluorescence assay (IFA) identification, purity testing, and whole-genome sequencing of each isolate. Additionally, the tissue culture infectious dose (TCID_50_) of each strain was determined, and viral dilutions were prepared based on the TCID_50_ results for virulence testing. The isolates were identified using anti-FAdV-4 monoclonal antibodies (Wei et al., 2022) through IFA (Wei et al., 2022). RT–PCR and PCR were used to detect common coinfecting avian viruses, such as Newcastle disease virus (NDV) (Feng et al., 2019), infectious bronchitis virus (IBV) (Wu et al., 2021), avian influenza virus (AIV) (Ip et al., 2016), laryngotracheitis virus (LTV) (Xu et al., 2017), reovirus (RLV) (Ye et al., 2020), avian leukosis virus (ALV) (Bi et al., 2014), reticuloendotheliosis virus (REV) (Aly et al., 1993), *Mycoplasma* (Xie et al., 2004), and infectious bursal disease virus (IBDV) (Su et al., 1997), to eliminate the possibility of contamination.

### 2.5 TCID_50_ determination for the isolates

The TCID_50_ was measured for each isolated strain separately. The viral stock was diluted in a 10-fold gradient (10^−1^ to 10^−10^) using DMEM/F12 medium and then inoculated into a 96-well culture plate with a monolayer of CEL cells. Each well received 0.1 mL of the dilution, with 8 replicate wells for each dilution, and blank cell control wells were included. After 1 h of infection, 0.1 mL of DMEM/F12 culture medium containing 5% FBS was added to each well. The cells were incubated at 37°C with 5% CO_2_ for 8 days. Cytopathic effects (CPEs) were observed in each cell well, and the effects in the wells with and without CPEs were recorded. The TCID_50_ was calculated using the Reed-Muench method.

### 2.6 Determination of the pathogenicity of the isolates from SPF chickens

The pathogenicity of 10 isolates of FAdV-4 was evaluated in 4-week-old SPF chickens. The chickens were divided into 9 groups based on the inoculum dose (10^0^ TCID_50_, 10^1^ TCID_50_, 10^2^ TCID_50_, 10^3^ TCID_50_, 10^4^ TCID_50_, 10^5^ TCID_50_, 10^6^ TCID_50_, 10^7^ TCID_50_, and 10^8^ TCID_50_), and 10 birds in each group received intramuscular injections. Additionally, a control group of chickens received injections of an equal volume of PBS solution. Each group of chickens was individually housed in separate SPF chicken isolators. The chickens were monitored for signs of illness or mortality each day for 14 days following inoculation. The incidence and mortality rates were then calculated based on the collected data. Survival curves were plotted using the Prism 8.0 software package (GraphPad Software Inc., San Diego, USA). FAdV-4 was reisolated from the liver samples of deceased chickens in each isolate group. The Fiber-1 and Fiber-2 encoding genes were amplified using primers 27, 28, and 29, as detailed in Supplementary Table S1, followed by sequencing for identification.

### 2.7 Whole-genome sequencing and bioinformatics analysis

DNA extracted from the isolates using the EasyPure Viral DNA/RNA Kit (TransGen Biotech) served as the template for PCR amplification, and a total of 56 primer pairs designed according to Luan et al. (2020) were used for whole-genome sequencing of FAdV-4 (Supplementary Table S1). Each position of the entire genome was sequenced independently 2–6 times to ensure the accuracy of the sequencing results. The PCR protocol was the same as that described previously. The PCR products were subjected to 1.2% agarose gel electrophoresis and visualized with GelRed dye. The PCR products were cloned and inserted into the pMD18-T vector and sent to Sangon Biotech Company for sequencing. The complete sequences were manually assembled using the SeqMan program (version 5.01) from the Lasergene software package (DNASTAR, Madison, WI). The entire gene sequence was submitted to the GenBank database, and a sequence accession number was obtained.

The complete nucleotide sequences of the isolates were aligned with sequences from 51 reference strains of FAdV-A to FAdV-E obtained from the GenBank database via the ClustalW alignment method. Neighbor-joining analysis was performed to construct a phylogenetic tree using MEGA 11 software (version 11) for molecular evolutionary genetics analysis. The evolutionary distances were calculated using the maximum composite likelihood method, and the tree was validated with 500 bootstrap replicates. Using MegAlign software (DNASTAR, Madison, WI), we analyzed the similarities/differences in nucleotide and amino acid sequences encoding the major structural proteins Hexon, Penton base, Fiber-1 and Fiber-2 from 10 isolates with novel genotype FAdV-4 strains (AH-F19, SD1601, JS07, HLJFAd15, CH/SXCZ/2015, and JSJ13), as well as nonvariant strains (B1-7, ON1, KR5, and MX-SHP95 strains). Characteristic amino acid mutation sites were identified.

To investigate whether strain GX2019-014 is involved in recombination events between the highly pathogenic novel genotype FAdV-4 and nonvariant FAdV-4 strains, we employed the RDP, GENECONV, BootScan, MaxChi, 3Seq, SiScan, Chimaera, and LARD analyses available in the RDP4 software. Analysis was conducted using preset data processing options, and the highest acceptable *p* value was set to 0.05. Additionally, SimPlot and BootScan analysis methods available in SimPlot software (version 3.5.1) were employed for sequence alignment analysis.

## 3 Results

### 3.1 Isolation and identification of FAdV-4

From 2019 to 2020, a total of 10 strains of FAdV-4 were isolated from the liver and kidney tissues of chickens in Guangxi Province, China. Among these strains, 4 strains were from Nanning city (GX2019-010, GX2019-011, GX2019-012, and GX2019-016), 4 strains were from Baise city (GX2019-013, GX2019-014, GX2019-015, and GX2019-018), and 2 strains were from Yulin city (GX2019-017 and GX2020-019). After three rounds of plaque purification, all 10 isolates replicated well in CEL cells, with observable changes in cell morphology, including rounding and detachment. When CEL cells were inoculated at an MOI of 0.01, the cytopathic effect area reached over 80% between 72 and 96 h postinoculation. The harvested cell culture supernatant had a TCID_50_ ranging from 10^8.0^ to 10^9.3^ TCID_50_/mL.

**Figure 1:**
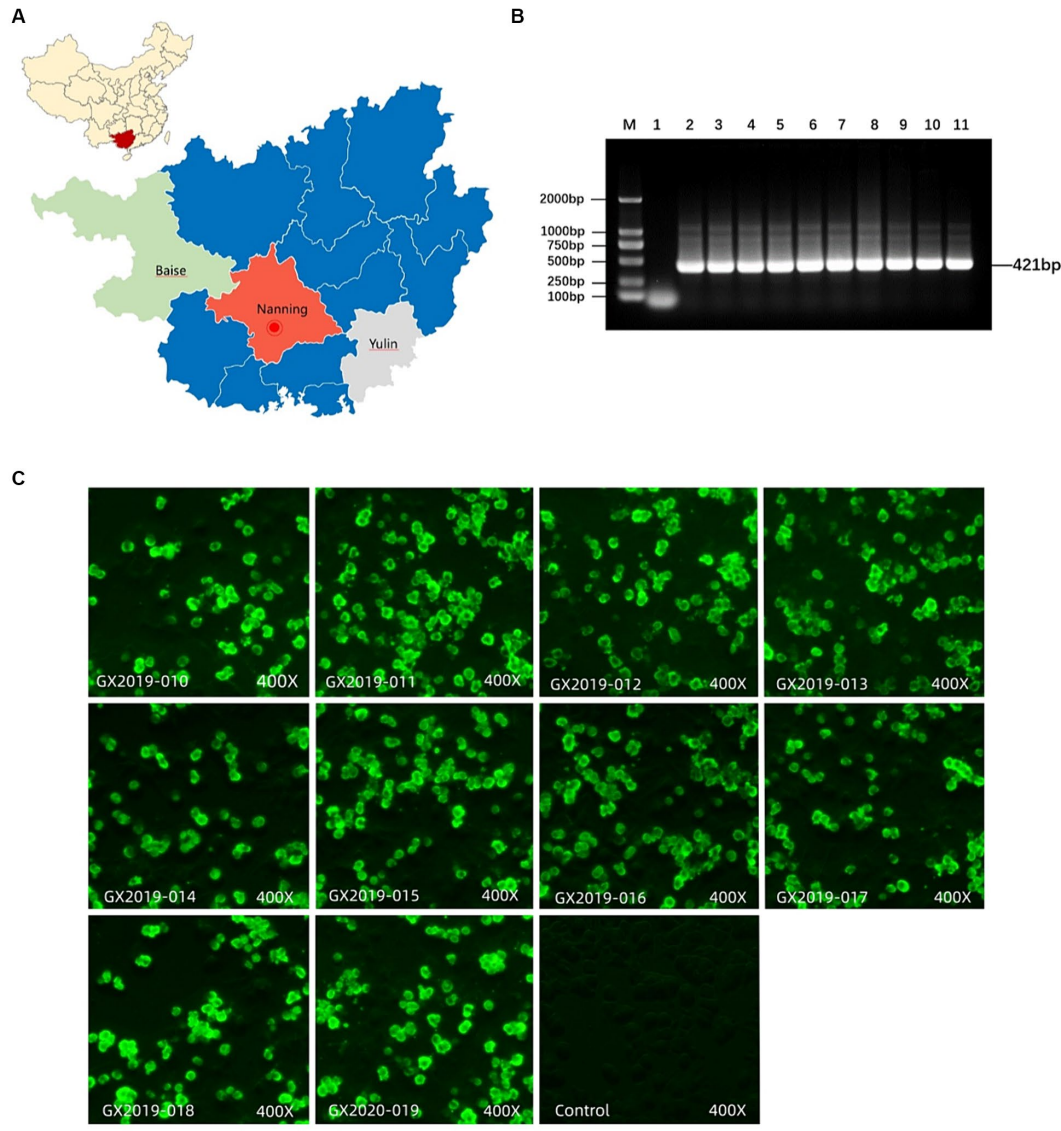
Isolation and identification of FAdV-4. **(A)** Location of sample collection. **(B)** Detection of FAdV-4 virus by PCR. M: DL2000 DNA marker; Lane 1: negative control; lanes 2–11: GX2019-010, 011, 012, 013, 014, 015, 016, 017, 018, and GX2020-019 PCR amplification products, respectively. **(C)** Identification of isolates using FAdV-4 Fiber-2 monoclonal antibodies via IFA.

Using indirect immunofluorescence analysis (IFA) with FAdV-4-Fiber2 monoclonal antibody, all 10 isolates showed positive reactions. PCR analysis was performed to exclude contamination by common coinfecting viruses, such as NDV, IBV, AIV, LTV, RLV, ALV, mycoplasma, REV, and IBDV. The isolation and purification results are shown in Figure 1.

### 3.2 Pathogenicity of the isolates on SPF chickens

**Figure 2:**
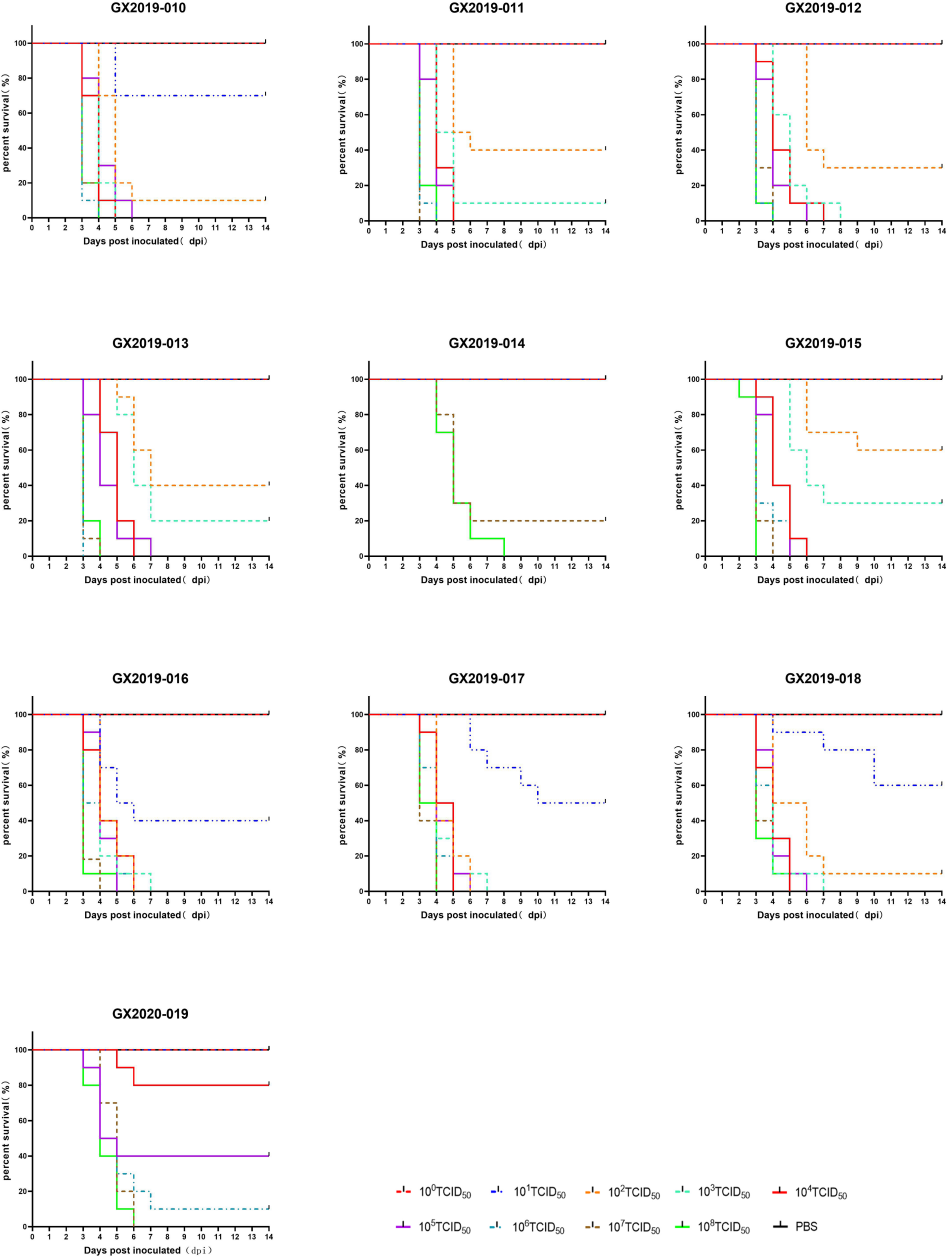
Survival curves of SPF chickens infected with different doses of the isolates.

Different doses of the isolates were intramuscularly injected into 4-week-old SPF chickens to cause infection. In terms of lethality, GX2019-010, GX2019-016, GX2019-017, and GX2019-018 exhibited the greatest virulence, with a dose of 10^2^ TCID_50_ resulting in 90 to 100% mortality in SPF chickens. The next highest virulence levels were observed for GX2019-011, GX2019-012, GX2019-013, and GX2019-015, in which a dose of 10^3^ TCID_50_ caused 70 to 100% mortality in SPF chickens. The virulence of GX2020-019 was weaker, as a mortality rate of 60% was observed when the chickens were inoculated with 10^5^ TCID_50_ of the virus. The virulence of GX2019-014 was the weakest, as experimental chickens inoculated with doses ranging from 10^0^ TCID_50_ to 10^6^ TCID_50_ showed no mortality, whereas doses of 10^7^ TCID_50_ and 10^8^ TCID_50_ resulted in mortality rates of 80 and 100%, respectively (Figure 2; Table 2).

**Table 2:** Lethality of different doses of isolates on SPF chickens.

Strain	Mortality rate (death number/total number)								
	10^8^ TCID_50_	10^7^ TCID_50_	10^6^ TCID_50_	10^5^ TCID_50_	10^4^ TCID_50_	10^3^ TCID_50_	10^2^ TCID_50_	10^1^ TCID_50_	10^0^ TCID_50_
GX2019-010	10/10	10/10	10/10	10/10	10/10	10/10	9/10	3/10	0/10
GX2019-011	10/10	10/10	10/10	10/10	10/10	9/10	6/10	0/10	0/10
GX2019-012	10/10	10/10	10/10	10/10	10/10	10/10	7/10	0/10	0/10
GX2019-013	10/10	10/10	10/10	10/10	10/10	8/10	6/10	0/10	0/10
**GX2019-014**	**10/10**	**8/10**	**0/10**	**0/10**	**0/10**	**0/10**	**0/10**	**0/10**	**0/10**
GX2019-015	10/10	10/10	10/10	10/10	10/10	7/10	4/10	0/10	0/10
GX2019-016	10/10	10/10	10/10	10/10	10/10	10/10	10/10	6/10	0/10
GX2019-017	10/10	10/10	10/10	10/10	10/10	10/10	10/10	5/10	0/10
GX2019-018	10/10	10/10	10/10	10/10	10/10	10/10	9/10	4/10	0/10
GX2020-019	10/10	10/10	10/10	6/10	2/10	0/10	0/10	0/10	0/10

**Figure 3:**
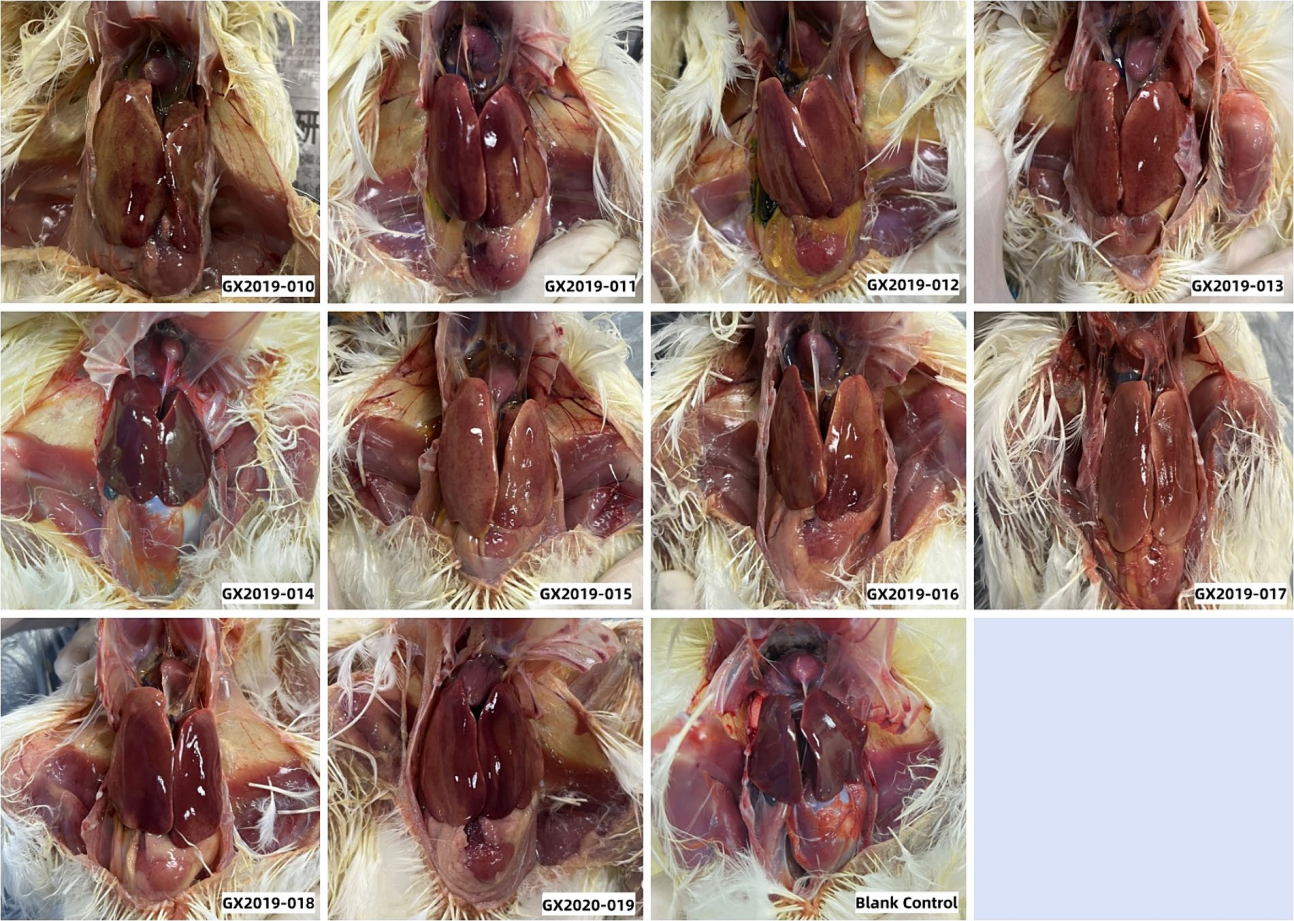
Gross lesions observed in 4-week-old SPF chickens infected with isolates at a dosage of 10^6^ TCID_50_.

Autopsies were performed on the deceased chickens and those surviving 14 days postinoculation. All 10 isolates from the dead chickens exhibited typical gross lesions of HHS caused by FAdV-4. These lesions included amber-colored fluid accumulation in the pericardium, significant hepatomegaly, jaundice, and hemorrhagic spots. Up to a dose of 10^6^ TCID_50_, no differences were detected between chickens inoculated with the GX2019-014 strain and those in the blank control group (Figure 3). The results of viral reisolation indicated that the virus isolated from the deceased chickens matched the inoculated isolate.

### 3.3 Phylogenetic analysis and molecular characterization of the FAdV-4 strains

**Figure 4:**
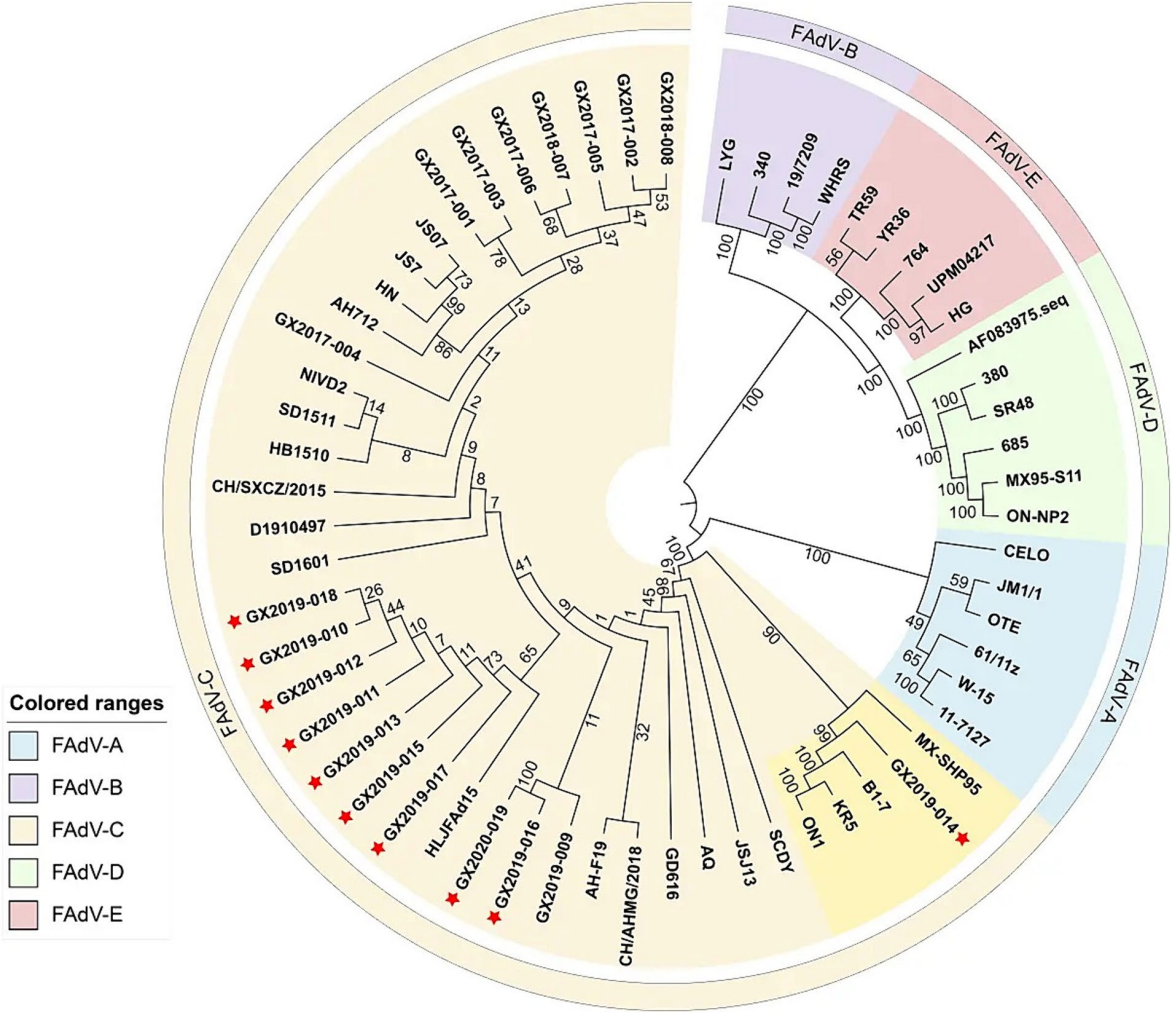
Phylogenetic analysis of complete nucleotide sequences based on FAdVs. The phylogenetic tree was constructed using the neighbor-joining method in MEGA 11 software, with 500 bootstrap replicates, and evolutionary distances were computed using the maximum composite likelihood method. The strains isolated in this study are indicated by red asterisks.

The complete genome lengths of the 10 FAdV-4 strains obtained in this study ranged from 43,714 to 43,721 bp, with a G+C ratio between 54.87 and 54.98%, and all the genomes encoded 43 open reading frames (ORFs). The complete genome sequences have been uploaded to GenBank and assigned accession numbers, as detailed in Table 3. Phylogenetic analysis based on the complete genomes indicated that all 10 strains were FAdV-C strains. Their genomic homologies with FAdV-A, FAdV-B, FAdV-D, and FAdV-E ranged from 55.1 to 55.3%, 55.9 to 58.4%, 55.7 to 56.2%, and 56.9 to 57.3%, respectively, suggesting distant relationships. Notably, compared to the newly emerged highly pathogenic FAdV-4 strains in China (such as AH-F19, JS07, SD1601, HLJFAd15, CH/SXCZ/2015, and JSJ13), GX2019-014 shows a closer genetic relationship to virulent strains from other countries (such as MX-SHP95, ON1, KR5, and B1-7), as illustrated in Figure 4.

**Table 3:** Complete genomic information of the isolates and reference strains.

Name of the isolate	Genome length	GenBank accession No.	GC content	Species	Serotypes	Country
GX2019-010	43,719 bp	MW439040	54.87%	C	4	China
GX2019-011	43,721 bp	MW439041	54.87%	C	4	China
GX2019-012	43,719 bp	MW439042	54.87%	C	4	China
GX2019-013	43,721 bp	MW439046	54.87%	C	4	China
GX2019-014	43,715 bp	MW448476	54.98%	C	4	China
GX2019-015	43,721 bp	ON665754	54.87%	C	4	China
GX2019-016	43,714 bp	MW439043	54.88%	C	4	China
GX2019-017	43,721 bp	MW439044	54.87%	C	4	China
GX2019-018	43,721 bp	MW439045	54.87%	C	4	China
GX2020-019	43,714 bp	OP378126	54.88%	C	4	China
GX2017-001	43,725 bp	MN577977	54.87%	C	4	China
GX2017-002	43,725 bp	MN577978	54.85%	C	4	China
GX2017-003	43,723 bp	MN577979	54.87%	C	4	China
GX2017-004	43,723 bp	MN577980	54.87%	C	4	China
GX2019-005	43,722 bp	MN577981	54.87%	C	4	China
GX2019-006	43,718 bp	MN577982	54.87%	C	4	China
GX2018-007	43,719 bp	MN577983	54.87%	C	4	China
GX2018-008	43,726 bp	MN577984	54.87%	C	4	China
GX2018-009	43,726 bp	MN577985	54.87%	C	4	China
HLJFAd15	43,720 bp	KU991797.1	54.87%	C	4	China
CH/SXCZ/2015	43,721 bp	KU558762.1	54.87%	C	4	China
HB1510	43,721 bp	KU587519.1	54.87%	C	4	China
JSJ13	43,755 bp	KM096544.1	54.88%	C	4	China
NIVD2	43,719 bp	MG547384	54.87%	C	4	China
AH-F19	43,719 bp	MN781666	54.87%	C	4	China
CH/AHMG/2018	43,721 bp	MN606303.1	54.87%	C	4	China
D1910497	43,717 bp	MW711380.1	54.88%	C	4	China
GD616	43,259 bp	MW509553.1	54.97%	C	4	China
JS07	43,723 bp	KY436519	54.87%	C	4	China
AQ	43,723 bp	KY436520	54.87%	C	4	China
HN	43,724 bp	KY379035	54.87%	C	4	China
AH712	43,275 bp	KY436522	54.87%	C	4	China
SCDY	43,677 bp	MK629523	54.84%	C	4	China
SD1601	43,723 bp	MH006602	54.87%	C	4	China
SD1511	43,722 bp	MF496037	54.87%	C	4	China
D1910497	43,717 bp	MW711380	54.88%	C	4	USA
MX-SHP95	45,641 bp	KP295475	54.73%	C	4	Mexico
ON1	45,667 bp	GU188428.1	54.63%	C	4	Canada
KR5	45,810 bp	HE608152.1	54.63%	C	4	Austria
B1-7	45,622 bp	KU342001.1	54.64%	C	4	India
CELO	43,804 bp	U46933.1	54.30%	A	1	Austria
61/11z	43,854 bp	KX247012.1	54.30%	A	1	Poland
JM1/1	43,809 bp	MF168407.1	54.31%	A	1	Japan
11–7,127	43,795 bp	MK572848.1	54.30%	A	1	Japan
OTE	43,816 bp	MK572847.1	54.25%	A	1	Japan
W-15	43,849 bp	KX247011.1	54.31%	A	1	Poland
340	45,781 bp	NC021211.1	56.52%	B	5	Ireland
WHRS	45,734 bp	OM836676.1	56.55%	B	5	China
19/7209	45,794 bp	OK283055.1	56.66%	B	5	Hungary
LYG	45,781 bp	MK757473.1	5,758%	B	5	China
AF083975	45,063 bp	AF083975.2	53.78%	D	9	Canada
SR48	43,632 bp	KT862806.1	53.33%	D	2	Austria
ON-NP2	45,193 bp	KP231537.1	54.07%	D	11	Canada
MX95-S11	44,326 bp	KU746335.1	53.69%	D	11	Mexico
380	43,347 bp	KT862812.1	53.27%	D	11	Britain
685	44,336 bp	KT862805.1	53.29%	D	2	Britain
HG	44,055 bp	GU734104.1	57.92%	E	8	Canada
UPM04217	44,072 bp	KU517714.1	57.93%	E	8b	Malaysia
764	43,666 bp	KT862811.1	57.81%	E	8b	Britain
TR59	43,287 bp	KT862810.1	57.94%	E	8a	Japan
YR36	43,525 bp	KT862809.1	57.78%	E	7	Japan

Compared to the nonvariant FAdV-4 strains B1-7, ON1, KR5, and MX-SHP95, the 10 isolates displayed extended GA sequences within the GA repeat region located between the PX and PVI genes, as well as longer TC repeat sequences within the TC repeat region situated between the protease and DBP genes and 15 to 99 additional base pairs in the ORF19A region. In addition, 3 base pairs in the coding region of the DNApol protein were deleted, and 1966 base pairs in ORF19, ORF27, and ORF48 were deleted. In contrast to the nonpathogenic ON1 strain, the coding sequence of Fiber-2 exhibited an insertion of 15 additional base pairs, leading to the incorporation of 5 extra amino acids (ENGKP). These changes are consistent with the new genotype FAdV-4 isolated in China after 2015. However, other molecular features of the new genotype FAdV-4 in China were observed in only 9 of the isolates (strain GX2019-014 was the exception). These changes included the insertion of 10 base pairs in the tandem repeat sequence (TR-B); the insertion of 3 base pairs each in the coding regions of ORF2, ORF22, and the 52/55 K protein; the deletion of 3 base pairs, resulting in the elimination of one histidine residue within the coding region at the end of Fiber-1; the deletion of 5 base pairs in the noncoding region between ORF30 and ORF17; and the insertion of 3 base pairs in the noncoding region after ORF43.

**Figure 5:**
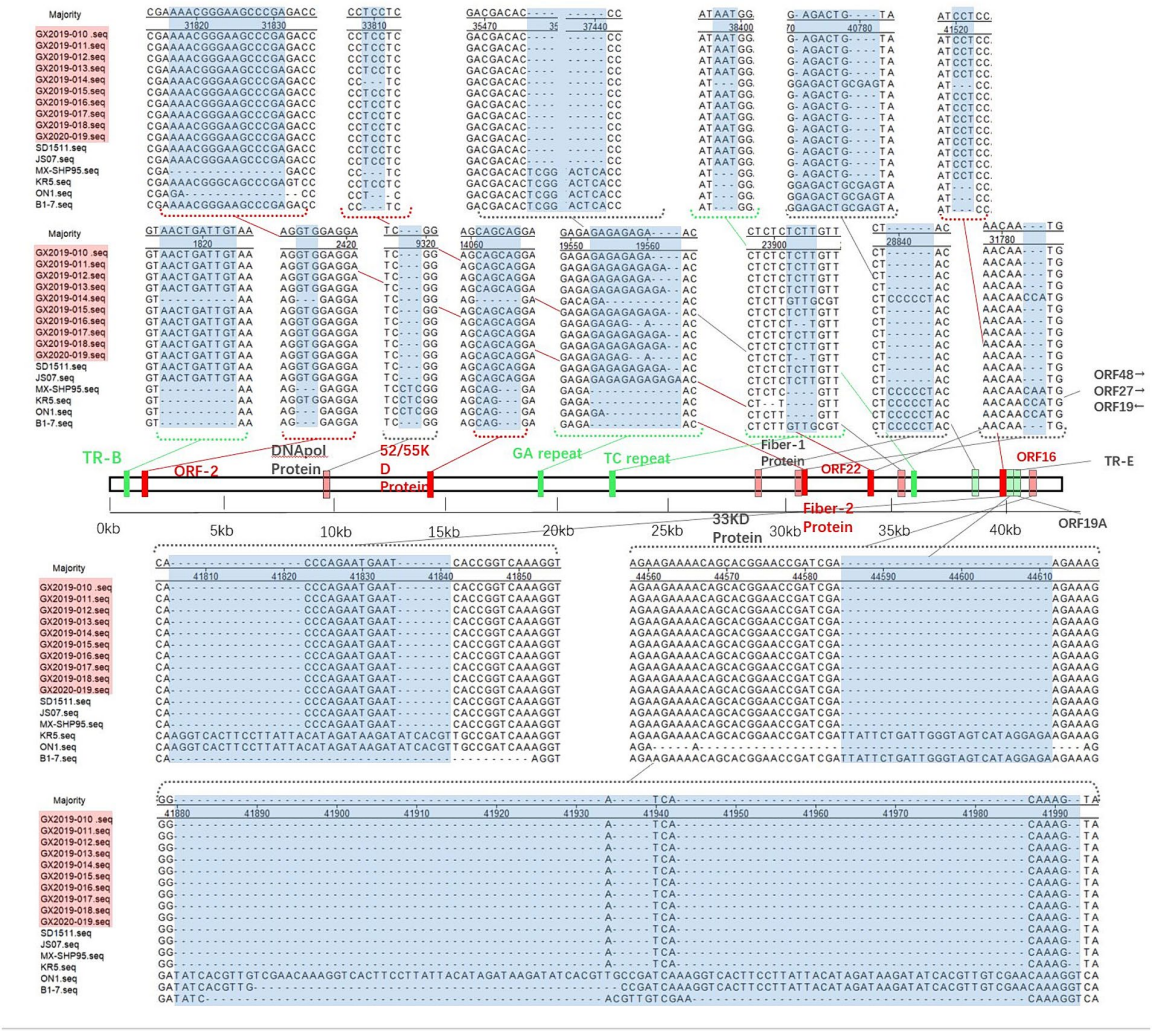
Insertion/deletion nucleotide positions in the whole-genome sequence of the isolates. The blue area represents regions of observed changes. The red area indicates the inserted coding sequence (CDS) regions. The light red area indicates the deleted CDS regions. The green area indicates inserted non-CDS regions. The light green area indicates deleted non-CDS regions.

The amino acid sequences of the primary structural proteins Penton base, Hexon, Fiber-2, and Fiber-1 in the 9 isolates, excluding strain GX2019-014, are highly homologous to those of the highly pathogenic novel genotype strain (99.9% ~ 100%). Compared to those of nonvariant strains, their homologies ranged from 94.8 to 99.7%, with 5 to 7, 12 to 13, 19 to 21, and 22 to 28 amino acid variation sites, respectively. In particular, the Penton base amino acid sequence of strain GX2019-014 has only 2 variation sites, unlike other newly emerged highly pathogenic strains, which exhibit variations at positions 45 (G to D), 356 (A to V), 370 (Q to P), 426 (I to V), and 485 (S to T). A similar pattern was observed for both the Fiber-1 and Fiber-2 proteins. The new highly pathogenic strains exhibited 7 variation sites in the Fiber-1 amino acid sequence at positions 28 (I to S), 122 (D to N), 199 (T to V), 254 (I to L), 265 (Q to H), 266 (E to D), and 311 (R to H) and 15 variation sites in the Fiber-2 amino acid sequence at positions 219 (G to D), 261 (S to T), 306 (H to N), 307 (P to A), 319 (V to I), 324 (F to V), 334 (T to A), 343 (N to L), 380 (A to T), 391 (S to T), 400 (A to G), 406 (S to I), 413 (T to S), 439 (D to E), 459 (A to N), and 418 (V to L). Interestingly, these variations are absent in strain GX2019-014 (refer to Figure 5; Table 4). Notably, the amino acids at positions 219 and 380 of the Fiber-2 protein in strain GX2019-014 differ from those in the pathogenic FAdV-4 strains but are identical to those in known nonpathogenic strains (B1-7, KR5, and ON1) (refer to the brown area in Table 4).

**Table 4:** Amino acid variation sites of Hexon, Penton base, Fiber-1, and Fiber-2.

Genes	Strains	Mutant sites																	
Penton		**42**	**45**		**193**	**356**	**370**	**426**		**486**									
	GX 201 9–010	P	D		I	V	P	V		T									
	GX 201 9–011	•	•		•	•	•	•		•									
	GX 201 9–012	•	•		•	•	•	•		•									
	GX 201 9–013	•	•		•	•	•	•		•									
	GX 201 9–014	•	**G**		•	**A**	**Q**	**I**		**S**									
	GX 201 9–015	•	•		•	•	•	•		•									
	GX 201 9–016	•	•		•	•	•	•		•									
	GX 201 9–017	•	•		•	•	•	•		•									
	GX 201 9–018	•	•		•	•	•	•		•									
	GX 201 9–019	•	•		•	•	•	•		•									
	SD1511	•	•		•	•	•	•		•									
	JSO7	•	•		•	•	•	•		•									
	MX-SHP95	•	G		V	A	Q	I		S									
	ON1	S	G		V	A	Q	I		S									
	KR5	S	G		V	A	Q	I		S									
	B1--7	•	G		•	A	Q	I		S									
Hexon		**164**	**188**		**193**	**195**	**238**	**240**		**243**	**263**	**264**	**402**	**410**	**574**	**795**	**797**	**842**	
	GX 201 9–010	S	R		R	Q	D	T		N	I	V	A	A	I	Q	P	A	
	GX 201 9–011	•	•		•	•	•	•		•	•	•	•	•	•	•	•	•	
	GX 201 9–012	•	•		•	•	•	•		•	•	•	•	•	•	•	•	•	
	GX 201 9–013	•	•		•	•	•	•		•	•	•	•	•	•	•	•	•	
	GX 201 9–014	•	•		•	•	•	•		•	•	•	•	•	•	•	•	•	
	GX 201 9–015	•	•		•	•	•	•		•	•	•	•	•	•	•	•	•	
	GX 201 9–016	•	•		•	•	•	•		•	•	•	•	•	•	•	•	•	
	GX 201 9–017	•	•		•	•	•	•		•	•	•	•	•	•	•	•	•	
	GX 201 9–018	•	•		•	•	•	•		•	•	•	•	•	•	•	•	•	
	GX 201 9–019	•	•		•	•	•	•		•	•	•	•	•	•	•	•	•		
	SD1511	•	•		•	•	•	•		•	•	•	•	•	•	•	•	•	
	JSO7	•	•		•	•	•	•		•	•	•	•	•	•	•	•	•	
	MX-SHP95	T	•		Q	E	N	A		E	M	I	Q	T	V	•	A	G	
	ON1	T	I		Q	E	N	A		E	M	I	•	T	V	•	A	G	
	KR5	T	I		Q	E	N	A		E	M	I	•	T	•	E	•	G	
	B1--7	S	I		Q	E	N	A		E	M	I	•	T	V	•	A	G	
Fiber-1		**14**	**28**		**44**	**69**	**70**	**80**		**81**	**82**	**98**	**122**	**129**	**138**	**156**	**189**	**199**	
GX 201 9–010	A	S		R	G	S	--		--	--	D	N	A	A	R	D	V	
GX 201 9–011	•	•		•	•	•	--		--	--	•	•	•	•	•	•	•	
GX 201 9–012	•	•		•	•	•	--		--	--	•	•	•	•	•	•	•	
GX 201 9–013	•	•		•	•	•	--		--	--	•	•	•	•	•	•	•	
GX 201 9–014	•	**I**		•	•	•	G		G	G	•	**D**	•	•	•	•	**T**	
GX 201 9–015	•	•		•	•	•	--		--	--	•	•	•	•	•	•	•	
GX 201 9–016	•	•		•	•	•	-		--	--	•	•	•	•	•	•	•	
GX 201 9–017	•	•		•	•	•	--		--	--	•	•	•	•	•	•	•	
GX 201 9–018	•	•		•	•	•	--		--	--	•	•	•	•	•	•	•	
GX 201 9–019	•	•		•	•	•	--		--	--	•	•	•	•	•	•	•	
SD1511	•	•		•	•	•	•		--	--	•	•	•	•	•	•	•	
JSO7	•	•		•	•	•	•		--	--	•	•	•	•	•	•	•	
MX-SHP95	V	I		P	S	G	•		--	--	•	D	V	•	H	N	•	
ON1	V	I		P	S	G	•		--	--	•	D	V	•	H	N	T	
KR5	•	I		P	S	G	G		--	--	N	•	•	S	H	•	•	
B1--7	•	I		•	S	G	G		G	--	N	•	•	S	H	•	•	
	**207**	**254**		**265**	**266**	**313**	**332**		**334**	**377**	**386**	**404**	**431**	**434**				
GX 201 9–010	G	L		H	D	H	H		R	S	I	N	--	G				
GX 201 9–011	•	•		•	•	•	•		•	•	•	•	•	•				
GX 201 9–012	•	•		•	•	•	•		•	•	•	•	•	•				
GX 201 9–013	•	•		•	•	•	•		•	•	•	•	•	•				
GX 201 9–014	A	**I**		**Q**	**E**	**R**	•		•	•	•	•	H	A				

GX 201 9–015	•	•		•	•	•	•		•	•	•	•	•	•				
GX 201 9–016	•	•		•	•	•	•		•	•	•	•	•	•				
GX 201 9–017	•	•		•	•	•	•		•	•	•	•	•	•				
GX 201 9–018	•	•		•	•	•	•		•	•	•	•	•	•				
GX 201 9–019	•	•		•	•	•	•		•	•	•	•	•	•				
SD1511	•	•		•	•	•	•		•	•	•	•	•	•				
JSO7	•	•		•	•	•	•		•	•	•	•	•	•				
MX-SHP95	A	I		Q	E	R	Q		K	P	L	Y	N	•				
ON1	•	I		Q	E	R	Q		K	P	L	Y	H	S				
KR5	•	I		Q	E	R	Q		K	P	L	Y	H	S				
B1--7	•	I		•	•	R	•		•	•	L	•	--	•				
Fiber-2		**11–15**	**17**	**22**	**29**	**114**	**144**	**219**	**232**	**261**	**300**	**305**	**306**	**307**	**319**	**324**	**329**	**334**	**338**	GX 201 9–010	ENGKP	T	S	A	D	S	D	Q	T	T	A	N	A	I	V	L	A	N	GX 201 9–011	•	•	•	•	•	•	•	•	•	•	•	•	•	•	•	•	•	•	GX 201 9–012	•	•	•	•	•	•	•	•	•	•	•	•	•	•	•	•	•	•	GX 201 9–013	•	•	•	•	•	•	•	•	•	•	•	•	•	•	•	•	•	•	GX 201 9–014	•	•	•	•	A	A	**G**	•	**N**	•	•	**H**	**P**	**V**	**F**	•	**T**	•	GX 201 9–015	•	•	•	•	•	•	•	•	•	•	•	•	•	•	•	•	•	•	GX 201 9–016	•	•	•	•	•	•	•	•	•	•	•	•	•	•	•	•	•	•	GX 201 9–017	•	•	•	•	•	•	•	•	•	•	•	•	•	•	•	•	•	•	GX 201 9–018	•	•	•	•	•	•	•	•	•	•	•	•	•	•	•	•	•	•	GX 201 9–019	•	•	•	•	•	•	•	•	•	•	•	•	•	•	•	•	•	•	SD1511	•	•	•	•	•	•	•	•	•	•	•	•	•	•	•	•	•	•	JSO7	•	•	•	•	•	•	•	•	•	•	•	•	•	•	•	•	•	•	MX-SHP95	-----	•	Y	P	A	A	•	•	N	•	•	H	•	•	F	•	T	T	ON1	-----	•	•	P	•	•	G	E	S	I	S	H	P	V	F	V	T	T	KR5	•	S	•	•	•	•	G	E	S	I	S	H	P	V	F	V	T	T	B1--7	•	•	•	•	•	•	G	Q	S	•	•	•	•	•	•	•	•	•
		**343**	**344**	**346**	**378**	**380**	**391**	**393**	**400**	**403**	**405**	**406**	**413**	**427**	**435**	**439**	**453**	**459**	**478**	GX 201 9–010	L	N	A	T	T	T	P	G	E	S	I	S	I	S	E	A	N	L	GX 201 9–011	•	•	•	•	•	•	•	•	•	•	•	•	•	•	•	•	•	•	GX 201 9–012	•	•	•	•	•	•	•	•	•	•	•	•	•	•	•	•	•	•	GX 201 9–013	•	•	•	•	•	•	•	•	•	•	•	•	•	•	•	•	•	•	GX 201 9–014	**W**	•	V	•	**A**	**S**	•	**A**	•	•	**S**	**T**	V	•	**D**	•	**A**	**V**	GX 201 9–015	•	•	•	•	•	•	•	•	•	•	•	•	•	•	•	•	•	•	GX 201 9–016	•	•	•	•	•	•	•	•	•	•	•	•	•	•	•	•	•	•	GX 201 9–017	•	•	•	•	•	•	•	•	•	•	•	•	•	•	•	•	•	•	GX 201 9–018	•	•	•	•	•	•	•	•	•	•	•	•	•	•	•	•	•	•	GX 201 9–019	•	•	•	•	•	•	•	•	•	•	•	•	•	•	•	•	•	•	SD1511	•	•	•	•	•	•	•	•	•	•	•	•	•	•	•	•	•	•	JSO7	•	•	•	•	•	•	•	•	•	•	•	•	•	•	•	•	•	•	MX-SHP95	N	S	V	•	•	S	S	A	Q	•	S	T	V	•	D	•	A	V	ON1	N	S	•	A	A	S	•	A	Q	P	S	T	•	T	D	S	A	V	KR5	N	S	•	A	A	S	•	A	Q	•	S	T	•	T	D	S	A	V	B1--7	•	•	•	A	A	S	•	•	•	•	•	•	V	•	•	S	A	V

• indicates amino acid identity with the GX2019-010 strain at the same locus; - indicates amino acid deletion at the locus in each strain; red letters denote amino acid identity with nonvariant strains at the locus in the GX2019-014 strain; brown shading represents potential virulence determinants of FAdV-4 predicted in this study.

RDP4 and Simplot software analyses did not provide statistical evidence for a recombination event involving strain GX2019-014 between pathogenic and nonpathogenic strains.

## 4 Discussion

Since 2015, large-scale outbreaks of HHS have occurred in multiple poultry farming provinces in China. This syndrome is characterized by a significant accumulation of fluid with an amber hue in the pericardium, along with swelling, jaundice, and hemorrhagic spots appearing in the liver. The mortality rate among affected chicken flocks can reach as high as 20 to 80%. The immune organs of surviving chickens are often damaged, leading to immunosuppression, which reduces the resistance of the flock to other pathogenic microorganisms. The significant economic losses have sparked researchers’ attention to the pathogenesis and prevention methods of this disease, making HHS and its pathogen FAdV-4 research areas of intense interest in recent years. Researchers have successively isolated and purified the virus from various tissues, such as the liver and kidneys of deceased chickens, identifying the causative agent of this outbreak as a novel genotype of FAdV-4 (Zhao et al., 2015; Kai et al., 2019; Rashid et al., 2020; Feng et al., 2021). The pathogenic identity of these strains was also confirmed through animal regression experiments.

Previous FAdVs typically showed lower virulence and often presented as subclinical infections within chicken populations. The mortality rate among chickens carrying the virus is not typically high, and few laying hens show symptoms (Asthana et al., 2013). However, the mortality rate among diseased chickens increases during coinfections, particularly coinfections with immunosuppressive viruses such as IBDV and ALV (Zhao et al., 2015). Investigations into the current HHS outbreak in China revealed that the FAdV-4 strain within the FAdV-C species is significantly predominant rather than the predominant FAdV-D and FAdV-E strains that have been prevalent globally over the past decade; in addition, this FAdV-4 strain exhibits distinctly high pathogenicity (Jing et al., 2017; Niu et al., 2018; Qingye et al., 2023). The novel genotype FAdV-4 strain SD1511, which was inoculated intramuscularly at a dose of 3 × 10^3.2^ TCID_50_ into SPF chickens at 21, 7, and 35 days of age, resulted in mortality rates of 80, 93, and 100%, respectively (Mo et al., 2019). When administered via intramuscular injection at a dose of 10^2.5^ TCID_50_ to SPF chickens at 3 weeks of age, the GD616 strain resulted in a mortality rate of 100% (Yuan et al., 2020). When administered via intramuscular injection at a dose of 10^6^ TCID_50_, the JS07 and HN strains isolated from eastern China exhibited mortality rates of 80 and 100%, respectively (Kai et al., 2019).

This study was conducted during the HHS outbreak from 2019 to 2020 in the southwestern Chinese province of Guangxi, which includes the major poultry farming cities of Nanning, Yulin, and Baise. Tissue samples from chicks in commercial chicken farms and backyard chicken households were used to detect the pathogenic agents of common avian diseases. Samples that tested positive for FAdV-4 by PCR were selected for viral isolation and purification, resulting in the acquisition of 10 isolates. The tissue samples from which these 10 isolates originated showed no presence of other viruses or mixed infections with other serotypes of fowl adenovirus. Research has shown that the newly emerged highly pathogenic FAdV-4 is an age-dependent virus (Mo et al., 2019; Feng et al., 2021), with chickens younger than 60 days being more susceptible and resistance increasing as age progresses. For the study of Chinese newly emerging highly pathogenic FAdV-4 strains, animal models typically utilize SPF chickens aged 3–4 weeks (Zhao et al., 2015; Cui et al., 2020; Luan et al., 2020). We opted for the consistent use of 4-week-old SPF chickens, with the inoculation dosage as the sole variable for virulence testing, aiming to reflect differences in pathogenicity among various strains. This approach facilitates comparisons with other FAdV-4 strains in animal models of similar ages. The results revealed that the mortality rates of strains GX2019-010 to 013 and 015 to 018, which were administered via intramuscular injection at a dose of 10^3^ TCID_50_, were 70 and 100%, respectively; these rates were similar to those of other isolates in China. Strain GX2020-019 demonstrated moderate virulence, with mortality rates of 0, 20 and 60% at 10^3^ TCID_50_, 10^4^ TCID_50_ and 10^5^ TCID_50_, respectively. Of particular significance, the GX2019-014 strain is characterized as a wild-type low-virulence strain. Up to a dose of 10^6^ TCID_50_, experimental chickens inoculated with this strain showed no clinical symptoms, and postmortem examination revealed no pericardial effusion, liver swelling or jaundice, consistent with the control group. However, at doses of 10^7^ TCID_50_ and 10^8^ TCID_50_, the mortality rates were 80 and 100%, respectively.

The novel genotype of FAdV-4 exhibits common molecular characteristics, such as a deletion of 1966 base pairs at ORF19, ORF27, and ORF48; longer GA repeats; and a deletion of three base pairs in the coding region of the DNApol protein (Pan et al., 2018; Mo et al., 2019; Rashid et al., 2020; Yin et al., 2020; Wei et al., 2023). These findings have prompted researchers to focus on identifying the key genes responsible for enhancing the virulence of FAdV-4. Using reverse genetic systems, researchers recently reported that deleting the 1966 bp nucleotide fragment does not affect virulence (Pan et al., 2018). Research by Liu et al. (2020) ruled out the influence of the Hexon protein and Penton on the increase in virulence. Through the ExoCET method, Zheng et al. discovered that the high virulence of the strains is associated with the Fiber-2 and Hexon proteins (Zhang et al., 2018). Quan et al. (2021) used the CRISPR/Cas9 technique to edit the Fiber-2 gene, resulting in a decrease in virus virulence. Therefore, it is widely believed that the virulence gene of FAdV-4 resides within the Fiber-2 protein.

In this study, with the exception of GX2019-014, the complete genomic sequences of the isolates exhibited high homology with the novel genotype FAdV-4 strains in China. However, GX2019-014 clustered with the nonpathogenic strains KR5, B1-7, and ON1 on the same branch. Analysis of the main structural proteins Penton base, Hexon, Fiber-1, and Fiber-2 of the 10 isolates, along with pathogenic and nonpathogenic strains of FAdV-4, revealed that the GX2019-014 strain showed greater homology to nonpathogenic strains (shown in Supplementary Figure S1). The Chinese isolates of the novel genotype FAdV-4 exhibit molecular characteristics such as a 10-base insertion in TR-B; three-base insertions in the coding regions of ORF2, ORF22, and the 52/55 K protein; and a three-base deletion at the end of the coding region of Fiber-1, leading to the loss of an amino acid residue. However, these molecular features were not observed in the GX2019-014 strain. The results of RDP4 and Simplot software analyses exclude the possibility that strain GX2019-014 is a recombination between nonpathogenic and pathogenic strains. Considering the results of pathogenicity testing, the GX2019-014 strain is more likely a wild-type low-virulence strain. Before the highly pathogenic novel genotype FAdV-4 emerged, studies suggested that residues D219, T300, and T380 in Fiber-2 were associated with virulence (Marek et al., 2012; Vera-Hernández et al., 2016). After more high-pathogenicity novel genotype FAdV-4 whole-genome sequences were deposited in GenBank, Yin et al. conducted comparisons using known pathogenic and nonpathogenic strain proteins, ruling out the impact of T300 on virulence (Yin et al., 2020). Zhang et al. (2021) previously proposed that amino acid residue 188 of the Hexon protein is a key virulence determinant and that the R188I mutation leads to reduced virulence in highly pathogenic strains. However, subsequent validation studies by Wang et al. did not fully support this view (Wang et al., 2022). The GX2019-014 strain, a novel low-pathogenicity genotype, provides new data for analyzing key virulence genes through its whole-genome sequence. By comparing the amino acid residues of major structural proteins among known pathogenic strains, nonpathogenic strains, and the GX2019-014 strain, it was found that there are no amino acid residues exclusively present in the highly pathogenic FAdV-4 strains on the Hexon, Penton, or Fiber-1 proteins. The presence of the 188R residue in the Hexon protein is consistent with that in other highly pathogenic strains, suggesting that this residue is not a key virulence determinant. However, on the Fiber-2 protein, residues D219 and T380 are unique to highly pathogenic FAdV-4 strains, with T380 located within the Knob domain of the Fiber-2 protein. This residue may serve as a potential key virulence determinant, consistent with previous research results. Nevertheless, further validation through the establishment of a reverse genetic system is needed.

In summary, we isolated and purified 10 strains of the novel genotype FAdV-4 from Guangxi Province in southwestern China between 2019 and 2020. We conducted pathogenicity assessments and whole-genome sequencing on these strains. Among these strains, the GX2019-014 strain showed nonpathogenicity in muscle in 4-week-old SPF chickens inoculated with 10^6^ TCID_50_ virus, and its whole-genome homology was closer to that of nonpathogenic FAdV-4 strains. This strain is the first reported wild-type low-virulence strain of the novel genotype FAdV-4. Our work has enriched the research data on prevalent strains of FAdV-4 in Guangxi Province, an important poultry farming region in China. This finding contradicts the previous notion that the Chinese strains isolated from the novel genotype FAdV-4 are highly pathogenic strains, providing reference material for further investigation into the key virulence-associated genetic loci of the novel genotype FAdV-4.

## Data availability statement

The original findings obtained from the study have been incorporated into the article and/or Supplementary Material. Inquiries can be directed to the corresponding author.

## Ethics statement

The animal study was approved by the Animal Ethics Committee of the Guangxi Veterinary Research Institute. The study was conducted in accordance with the local legislation and institutional requirements.

## Author contributions

YW: Data curation, Methodology, Supervision, Writing – original draft. ZhiqX: Investigation, Resources, Software, Writing – original draft. ZhixX: Funding acquisition, Project administration, Resources, Writing – review & editing. XD: Writing – original draft. XL: Project administration, Resources, Visualization, Writing – original draft. LX: Investigation, Software, Validation, Writing – review & editing. QF: Formal analysis, Investigation, Writing – review & editing. YZ: Investigation, Resources, Writing – review & editing. SW: Investigation, Methodology, Resources, Writing – review & editing. HR: Investigation, Software, Writing – review & editing. LW: Investigation, Software, Writing – review & editing. SL: Resources, Writing – review & editing. ML: Investigation, Methodology, Validation, Writing – review & editing.
