# Supplementary figures and images for: Differences in the pathogenicity and molecular characteristics of fowl adenovirus serotype 4 epidemic strains in Guangxi Province, southern China
Source: Front Microbiol. 2024 Jun 27;15:1428958. doi: 10.3389/fmicb.2024.1428958 (PMC11236736; doi:10.3389/fmicb.2024.1428958)

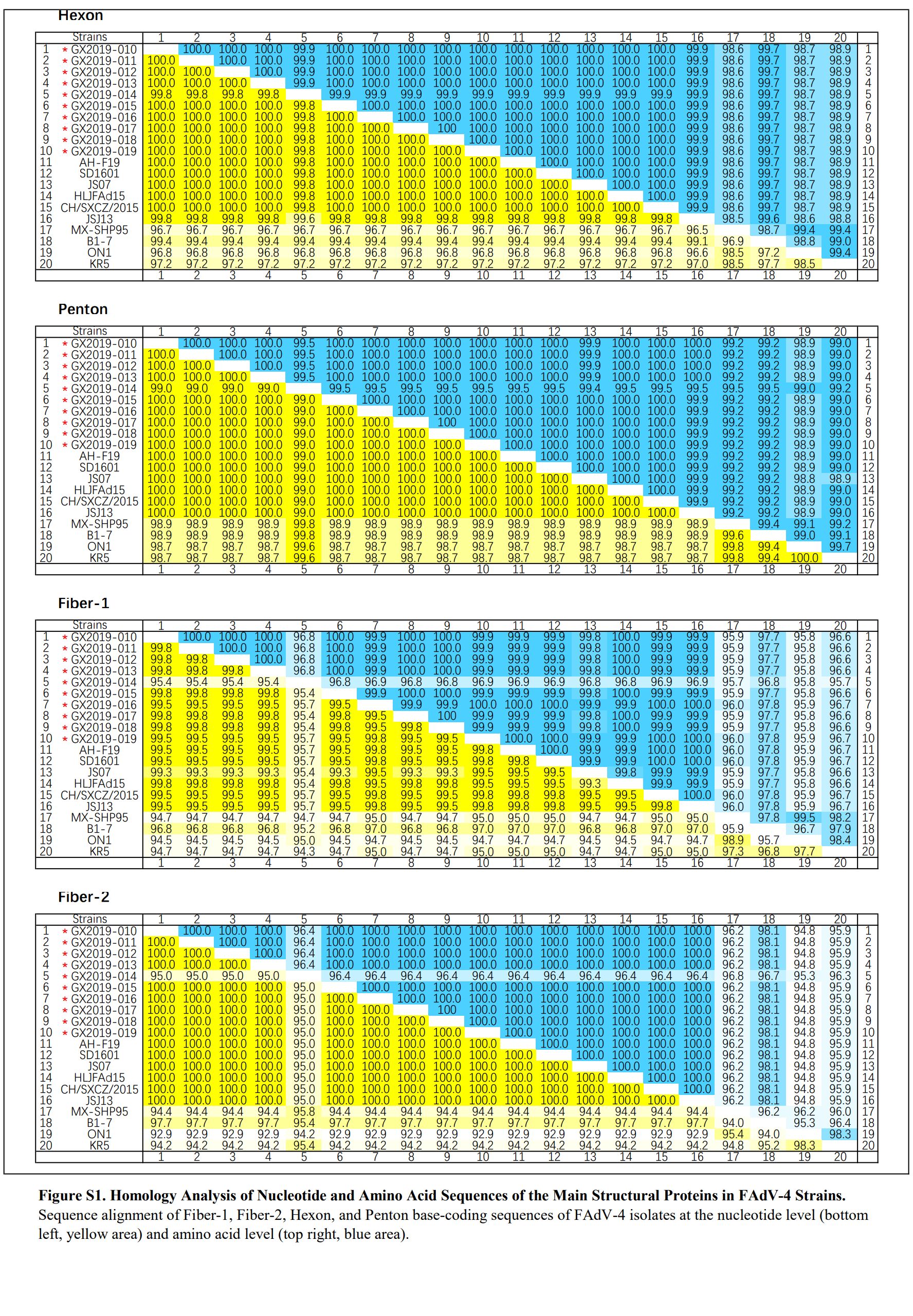

Supplement: Supplementary file 1 [file Image_1.JPEG]
