# Supplementary material for: Differences in the pathogenicity and molecular characteristics of fowl adenovirus serotype 4 epidemic strains in Guangxi Province, southern China
Source: Front Microbiol. 2024 Jun 27;15:1428958. doi: 10.3389/fmicb.2024.1428958 (PMC11236736; doi:10.3389/fmicb.2024.1428958)
Supplement: Supplementary file 2 [file Table_1.DOCX]

**Table S1. Primers used for whole-genome sequencing**

| No. | Forward primer（5'→3'） | Reverse primer（5'→3'） | length |
| --- | --- | --- | --- |
| 1 | CATCATCTTATATAACCGCGTCT | CCTCCTCGGATCGTGTCAT | 1213 |
| 2 | TTGAGCGAATCTTTACACCG | TCCACCATAGTTCCCTCCC | 1250 |
| 3 | GTGGATTGGCGGAATAGGG | TTGGGTTGACGAAGTAAGAGCA | 1358 |
| 4 | GCGTCCTTCTTGATCCTCG | CGTCCACCTGTCCTGCTTC | 1351 |
| 5 | ACTTGTTCGTCTTCGGGTGTC | CCTGTTCCTCCAACTGCCTC | 1375 |
| 6 | GGATGCTACTCTGGCGTTGT | CGACTCCTTTCGCTGGTG | 1437 |
| 7 | GCGAGTCTGAGGGAGAAATG | CCACAACGAGCAGCTAACG | 1398 |
| 8 | GGGGTGTTCGGTGTCGTA | TAGACATCATCACGCTTCACAA | 1367 |
| 9 | GGGCGTTGCTGAGCATTT | CACCTTACCGTCCGATTTCTA | 1404 |
| 10 | ATGAAACGCACAAAGACGG | AGACAAGTCGGGAGACATCG | 1530 |
| 11 | GATGGTATCGCTGTTGGAAGTC | GTCACCGACAGATCCGGATTAC | 910 |
| 12 | TATCGCTGTTGGAAGTCGC | AGAGGAGTCGTCGTGGGTC | 1364 |
| 13 | TCTATACGTGCTTTCGGTGGT | GCTGCGGGTTCAGTTTGA | 1507 |
| 14 | TATCGCTCGGGACAGGTAGT | GCCGTAGTCGTAGAAGGTGC | 1477 |
| 15 | TTGCTCCGCTTGTTCGTG | CGGTAAGTGTCCCTTAATAATGG | 1338 |
| 16 | GCGGAATCAGAGGGTCGGGACT | ATCGGGCACCGTCAGCAAGG | 1297 |
| 17 | AACGCTGCTCCCCTTTTA | GCCCGTAGTCAGGTCTCG | 1337 |
| 18 | ACAGACAGGACGGACCAGC | TGCGAACCTAGACGAAACG | 1414 |
| 19 | GAGATGGTGACGGAGGTG | CCAGTTTCTGTGGTGGTTG | 1786 |
| 20 | CCAACGCCACTACCAACT | GAAAGCGGTGACGAGGAT | 1670 |
| 21 | GTGGACCATCCCGTTCAGT | GCATCGAGCAGTGCGTGT | 1470 |
| 22 | TGTGCGGGTGCTTGTGGT | GCGAGGTAGGAGGCGACTAA | 1270 |
| 23 | CTGGTCGTCTTCTTCTTCGG | CAGAGTCGCTAGAGTGGCTAAA | 1434 |
| 24 | CGGTTACTATTCGGCAGATGG | GATAAGCCTCGATGGTTTCCT | 1256 |
| 25 | CCTTCCATCACGGTTTCG | TGCTCATCTGGTCCTCTTCC | 1330 |
| 26 | GCCCGAAATCTACAATCCC | ACCTCCCATCATGCCTCC | 1210 |
| 27 | CAGACCAACAGCCCTACGC | CGAGCACTTTGAGCACCC | 1268 |
| 28 | GCCACTAAGCAAGCCAACG | CCTGATCCACGAGCAAGGT | 1418 |
| 29 | ACGATGACTGGGAACTGGC | GGACAAATGGACGATCAATAAA | 1118 |
| 30 | CCGCTACACCCTTCTATGCT | CGGTCCCTTCTGTGATTGC | 1342 |
| 31 | CGGAGATTTGCGATTGTGAGT | TGACTCATCATGGGTGTGGC | 1456 |
| 32 | ACACTAACTTCCTCATTGACCCTC | TGTCTGTCTGAACCTGCCTACC | 1512 |
| 33 | ACGATGGCGTGATAGGCGGAGC | ATGAACCGTAGCCCCGCCCTTT | 1173 |
| 34 | ACTACCGAGATCAGCCTGAAGA | CAGACTAAGGGAAAGTTGGAGAA | 1225 |
| 35 | GAAATGCTTCCTCCTTCACG | AAGTTTATAGGGATCTCGGGTTA | 1239 |
| 36 | AACCCGAGATCCCTATAAACTT | TAGTGCCTGTCCATTTGCC | 1203 |
| 37 | TGGCAAATGGACAGGCACT | TTGATTCGGTGGAGGTCGT | 1292 |
| 38 | CCCACTACCGCTACCACCAC | ATCACGCTGACGCTCCTCC | 1356 |
| 39 | GTGTCATCCCTTACGGAAAC | GCACATGTCCCAGTAGGTC | 518 |
| 40 | GGAAGGCACAACATCCAAG | CTGTCTAGCTTTACGGAGC | 1202 |
| 41 | AGCATGAATCAACTCGGTGTC | CATCATCTTATATAACCGCGTCT | 945 |
| ●42 | GGATTGGCTGCAAAGTTC | CATCTGTAATGCGAGCAC | 844 |
| ●43 | GTGTGCCAAGACGACATTC | GAGTAAAAGCCGCATGCG | 702 |
| ●44 | CAGTTCGTCTGCACTCACG | CGAGTGGAAATACGTCTACG | 601 |
| ●45 | CCGATCTGTTTTCCTGTACG | CATCCGCTGGTGATATGTGC | 805 |
| ●46 | CAACGGCTGCAAATCCGC | GATAAAGTTGCGCCTCTG | 1113 |
| ●47 | CGTTACTGCTCCAGGAAAC | CGCTAATTGCCATTGCCAT | 1863 |
| ●48 | CCTCCCAAACATGGATGTC | GGATCTGCACTTACCAATGG | 1583 |
| ●49 | CTCTCCCTATCCTTACCTCAC | ATCCGTGGTGAGTGTACTAAG | 483 |
| ●50 | TCGCTAATTCTTCGACCATGC | GGAGTCGTGATACAGCAGGTT | 2279 |
| ●51 | TCTGGAACGGCACCGTGAAG | CTGAACCGGAGCAGCGTTAC | 1029 |
| ●52 | CTAAATCCAATTTCTCCCAAG | TCCTTTGGTAGGGACCGTGGG | 761 |
| ●53 | TTTTCACCGCTATAATCATT | TTCTGGGTGGGGTTGGA | 1094 |
| ●54 | CCACGGTATTCCATCCGAAGACT | TATTCCGTGACCTTTCTTCC | 1001 |
| ●55 | GACCTTGTCCTGTTTGTTTATTCAC | GTCTAGGAATATGCTTTTTTAGATC | 1076 |
| ●56 | GACCTTGTCCTGTTTGTTT | GGGACCACCACTCGCCATTTTTCTT | 1173 |

● indicates that the primers are designed for this project.
